# Is This Calculator Broken? Comparative Performance of Lung Nodule Risk Assessment Calculators in a High-Prevalence Cohort

**DOI:** 10.1016/j.chpulm.2025.100142

**Published:** 2025-01-31

**Authors:** Mateus Fernandes, Brett C. Bade

**Affiliations:** a Northwell Health, New Hyde Park, New York, NY; b Department of Medicine, Donald and Barbara Zucker School of Medicine at Hofstra/Northwell, New Hyde Park, New York, NY

Lung nodule management is an ongoing clinical challenge for pulmonologists. With implementation of lung cancer screening and commonplace acquisition of diagnostic chest CT scans, identification of lung nodules increasingly is common.^1^^,^^2^ Although most lung nodules are benign, a small portion are cancerous. Thus, the challenge for clinicians is to distinguish benign from malignant nodules (1) to ensure timely diagnosis of cancer in malignant nodules and (2) to minimize invasive procedures in patients with benign nodules.

Several guidelines exist to help clinicians navigate lung nodule management, including the Fleischner Society guidelines for incidental pulmonary nodules^3^ and Lung Imaging Reporting and Data System (Lung RADS) for screening-detected pulmonary nodules.^4^ For indeterminate pulmonary nodules, both American College of Chest Physicians^5^ and British Thoracic Society^6^ guidelines recommend estimating pretest probability of malignancy to inform follow-up strategies. Pretest probability may be estimated qualitatively (ie, clinical judgment) or quantitatively via validated risk calculators.^5^ Examples of risk-based calculators include the Mayo Clinic, Peking University (PKU), Veterans Affairs (VA), and Brock University models.^7^, ^8^, ^9^, ^10^ These calculators estimate pretest risk of malignancy by considering patient and radiologic characteristics (eg, tobacco smoking history, nodule size, lung location, and so on). In general, management guidelines recommend CT scan surveillance for low-risk nodules (< 5%-10% risk of malignancy), PET and CT imaging (and potentially nonsurgical biopsy) for moderate-risk nodules (5%-65% risk of malignancy), and resection or nonsurgical treatment for high-risk nodules (> 65%-70% calculated risk). Despite the availability of detailed clinical guidelines, routine nodule management often is discordant with risk assessment.^11^, ^12^, ^13^ In studies by Tanner and colleagues^11^^,^^12^ evaluating indeterminate pulmonary nodule management in community practices, clinician estimates of malignancy outperformed risk assessment tools. Although clinical intuition outperformed qualitative tools, rates of surgery were similar across the groups with low, intermediate, and high pretest risk of malignancy. Because surgery should be reserved for patients with high pretest risk of malignancy (to ensure low rates of invasive procedures in benign disease), it was unclear if community pulmonologists were unaware of guidelines, did not use risk assessment calculators, or believed that prediction models did not reflect their practice. For ease of clinical use, a risk assessment calculator should be readily available and should have fewer clinical criteria to incorporate. To give perspective, the number of clinical variables in the calculators referenced above are 4 for the VA model, 6 for the Mayo Clinic and PKU models, and 9 for the Brock model. Thus, much work must be done to ensure that clinicians align pretest risk of malignancy with nodule management strategies.

In this issue of *CHEST Pulmonary*, Pena and colleagues^14^ raise an important and unanswered question. Because risk assessment calculators are most effective when the study population matches the patient of interest, how effective are available lung nodule risk assessment tools in patients whose pretest risk of lung cancer is high? The author compared the performance of 4 risk models in a group of patients referred for pulmonary nodule evaluation. Each model’s testing characteristics were evaluated using both the American College of Chest Physicians and British Thoracic Society guidelines’ risk thresholds for ruling out (5% and 10%, respectively) and ruling in (65% and 70%, respectively). The cohort (N = 1,518 patients) predominately demonstrated incidental lung nodules (97%) and a smaller portion of screen-detected nodules (3%), and the prevalence of lung cancer was 72% (determined by histopathologic diagnosis or tumor board consensus). Although tumor board consensus is a suboptimal method to determine malignancy, comparing the models, the Mayo Clinic model showed the best discriminatory value with an area under receiver operating characteristic curve of 0.74. At the American College of Chest Physicians rule-out threshold (< 5% risk), the PKU model was the most sensitive (100%). At the American College of Chest Physicians rule-in threshold (65%), the PKU model was the most sensitive (80.8%) and the Brock model was the most specific (98%). The authors surmised that for the cohort in their study (at high risk of lung cancer), the PKU model was best for ruling in lung cancer, whereas the Brock was best for ruling out lung cancer. Notably, both the Brock and Mayo models were calibrated poorly, reflecting the lower prevalence of malignant nodules in the cohorts used to develop them. The PKU and VA models were calibrated better, likely reflecting the higher prevalence of malignancy in the study cohorts (53% and 54%, respectively), more closely matching the prevalence in the present study. Overall, each calculator exhibited moderate accuracy in this population with a high lung cancer prevalence. In sum, this study highlights a critical gap in the ability of current models to provide confident assessments for nodules that fall within higher-risk categories.

This study has several clinical implications. Most importantly, for clinicians using lung nodule risk assessment tools, it emphasizes the significance of aligning the right calculator with the right patient. In their review, Choi and colleagues^15^ used several clinical scenarios to demonstrate the variability of pretest malignancy estimates across different models. Thus, in clinical practice, using a model that aligns the patient characteristics with the development population is crucial. For instance, the high prevalence of lung cancer in this cohort (72%) may reflect patients being referred for biopsy to interventional pulmonology or thoracic surgery departments. With the very high rates of malignancy, it seems likely that the patients already had undergone a risk assessment, perhaps by another pulmonologist. In this group, it seems logical that the VA or PKU models (with higher prevalence of malignancy) would be more appropriate. By contrast, in patients evaluated in community practices after incidental imaging or lung cancer screening, the Mayo Clinic and Brock models (lung cancer prevalences of 23% and 5.5%, respectively) likely would be more appropriate.

In addition to model choice, this study also reiterates the evaluation strategy for lung nodules at high suspicion of malignancy. With only moderate diagnostic accuracy in patients with a high suspicion of malignancy, clinicians should recognize that if the nodule carries a higher baseline risk, then clinical judgment must play an important role. Clinician judgment is especially important in patients with conflicting clinical data. For example, seemingly negative biopsy findings in a patient at high pretest risk of malignancy cannot be assumed to be benign. Indeed, clinicians managing such patients must consider additional evaluation to ensure that diagnosis of malignant nodules is not delayed. Examples of additional evaluation may include short-interval follow-up imaging, repeat biopsy with navigational bronchoscopy or CT scan guidance, or surgical biopsy.

Finally, with imperfect risk assessment tools, it seems clear that additional strategies are needed for lung nodule assessment. Recent studies have highlighted the potential usefulness of biomarkers in indeterminate pulmonary nodules.^16^^,^^17^ With high negative predictive values (90%-98%), although lower specificity (44%), it seems likely that a strategy combining clinical and biomarker risk assessment may reduce the frequency of biopsies of benign nodules. Expanding the current models with so-called rule-in biomarkers may provide more accurate assessments and may reduce diagnostic uncertainty. In addition, radiomics and artificial intelligence also are making advances in risk prediction for indeterminate pulmonary nodules.^18^

In conclusion, the findings of this study underscore the importance of tailoring malignancy risk models to the population they intend to serve. Although current calculators remain useful for general practice, the high-prevalence setting poses unique challenges that require a more nuanced approach. Moving forward, dedicated calculators for high-risk settings or integration of biomarkers may bridge the current gap in diagnostic precision for lung nodules in high-prevalence populations.

## Financial/Nonfinancial Disclosures

The authors have reported to *CHEST Pulmonary* the following: B. C. B. is a site principal investigator for clinical trials for Delfi Diagnostics, Nucleix, and Biodesix. None declared (M. F.).
